# Mosquitoes and the Lymphatic Filarial Parasites: Research Trends and Budding Roadmaps to Future Disease Eradication

**DOI:** 10.3390/tropicalmed3010004

**Published:** 2018-01-04

**Authors:** Damilare O. Famakinde

**Affiliations:** Department of Medical Microbiology and Parasitology, College of Medicine of the University of Lagos, Idi-Araba, Lagos 100254, Nigeria; damfam.joe@gmail.com; Tel.: +234-703-330-2069

**Keywords:** mosquitoes, filarial parasites, vector–parasite system, lymphatic filariasis, eradication

## Abstract

The mosquito-borne lymphatic filariasis (LF) is a parasitic, neglected tropical disease that imposes an unbearable human scourge. Despite the unprecedented efforts in mass drug administration (MDA) and morbidity management, achieving the global LF elimination slated for the year 2020 has been thwarted by limited MDA coverage and ineffectiveness in the chemotherapeutic intervention. Moreover, successful and sustainable elimination of mosquito-vectored diseases is often encumbered by reintroduction and resurgence emanating from human residual or new infections being widely disseminated by the vectors even when chemotherapy proves effective, but especially in the absence of effective vaccines. This created impetus for strengthening the current defective mosquito control approach, and profound research in vector–pathogen systems and vector biology has been pushing the boundaries of ideas towards developing refined vector-harnessed control strategies. Eventual implementation of these emerging concepts will offer a synergistic approach that will not only accelerate LF elimination, but also augurs well for its future eradication. This brief review focuses on advances in mosquito–filaria research and considers the emerging prospects for future eradication of LF.

## 1. Introduction

Mosquitoes are unambiguously the most important vectors of infectious disease-causing agents that tremendously affect global health, with over half of the global human population at risk of exposure to mosquito-transmitted infections [1] and more than 1 billion cases of such infections reported each year [2]. *Brugia malayi* (Brugia), *Brugia timori* (Partono et al.), and, most importantly, *Wuchereria bancrofti* (Cobbold) are the mosquito-vectored filarial parasites causing human lymphatic filariasis (LF). They are transmitted by members of *Anopheles*, *Culex*, *Aedes*, *Mansonia,* and *Ochlerotatus* genera of mosquitoes, depending on the geographical location and biological peculiarities of each species [3]. Human LF is a neglected tropical disease (NTD) that presents with chronic disfiguring pathologies such as lymphoedema and elephantiasis (disfiguring swelling) of the legs, of the scrotum (hydrocoele) in males, and of the breasts and vulva in females [4], and the resulting deformities also generate severe psychosocial consequences including sexual disability [5] and mental depressive illness [6,7].

To address the unbearable disease scourge, the World Health Organization (WHO) launched the Global Programme to Eliminate Lymphatic Filariasis (GPELF) in the year 2000, with a commitment towards eliminating LF as a public health problem by 2020. While the GPELF also ensures morbidity management and disability prevention (MMDP), mass drug administration (MDA) has been the mainstay of the programme and generous drug donations from world-respected pharmaceutical companies (Eisai, GlaxoSmithKline, Johnson & Johnson, and Merck Sharp & Dohme [MSD]), have been facilitating wider global coverage of the MDA [8,9]. Depending on location and co-endemicity, the long-adopted WHO recommendations for MDA to eliminate LF are annual albendazole administration and annual combination therapy systems (using albendazole with ivermectin or with diethylcarbamazine) for at least five effective rounds. In May 2017, modifications to the existing recommendations were formulated to optimise human responses to the MDA regimens and accelerate the pace of the disease elimination. Annual triple-drug regimen (ivermectin + diethylcarbamazine + albendazole) and biannual albendazole were included, depending on the epidemiological and technical situations [10]. At the inception of GPELF, an estimated 120 million people were infected, of whom 40 million were seriously incapacitated and disfigured by the disease [11]. However, more recent data acknowledging a 13-year impact of the GPELF extrapolated that about 67 million people are now infected: 36 million presenting clinical illness, 19 million with genital hydrocoele, and 16 million with lymphoedema [12]. Currently, LF accounts for 2 million disease-adjusted life years (DALYs) [13], not including significant co-morbidity of mental illness commonly experienced by patients and caregivers [14].

Despite all efforts, global elimination of LF slated for the year 2020 is now practically unattainable, as 22 of the current 52 endemic countries requiring MDA have not commenced MDA in all of their endemic implementation units, and the suboptimal human response to current regimens post-MDA has surfaced [10,14,15]. The latter may have ensued due to the drug inactivity against adult worms [16] or inter- and intra-species variations causing a differential response to chemotherapy [17]; meanwhile, we cannot afford to overlook the possible emergence of drug-resistant microfilariae (mf) [18,19,20]. Beyond the walls of GPELF interventions, other pertinent factors are also pulling the strings. Successful control of mosquito-borne diseases is often challenged by reintroduction and resurgence emanating from new or residual human infections that are further disseminated by the vectors, especially when effective human vaccines are lacking. Although the application of insecticides has been largely buttressing mosquito control for many decades, toxicity to humans and emergence of insecticide-resistant traits among mosquito populations have been worrisome trends, and dampen the effectiveness of other control measures. This implies that even in the face of the new MDA adjustments, there is still a need to recreate momentum for vector control, if the goal is achieving sustainable elimination that will lead to disease eradication. This review focuses on the advances in the area of mosquito–filaria research and promising vector-based research initiatives that may unclog future eradication of LF.

## 2. The Mosquito–Filaria System: Past and Present Research

The present era of intense and expanding search for new strategies to abate vector-mediated pathogen transmission has extended a gradually-increasing priority for research on the mosquito–filaria system. Mosquitoes are equipped with physiological, immunological, and structural components that can preclude the establishment of filarial parasites [21,22,23,24]. Of particular interest is how the parasites adapt to the hostile vector environment and achieve transmission. Migratory timing and mechanical crossing of vector midgut barriers [23,25], as well as ingestion of glycogen granules and mitochondria of the thoracic muscle cells of the vectors [26,27,28], constitute some part of the physical strategies employed by filarial parasites.

Setting aside the physical and macromolecular adaptation mechanisms, recent post-genomic approaches—transcriptome analysis and proteomic profiling—on *B. malayi* provide increasing opportunity to identify and interrogate genes or proteins expressed during filarial parasite life-stages, serving as important tools in understanding the molecular underpinnings of the biological nature of filarial worms. In most molecular studies, *Aedes aegypti* (Linnaeus) and *B. malayi* are aptly used as a model system because of their easy adaptability and maintenance in the research laboratory [17,25]. Exsheathment of the ingested mf within the mosquito is required for morphological transformation of the parasites into the first-stage larvae (L1), and may occur in the mosquito midgut or haemocoel [23,29]. The assessed transcriptome and proteome of *B. malayi* mf showed upregulated transcripts encoding serpin (serine protease inhibitor), endochitinases (e.g., BmCHT1), proteases (e.g., metalloprotease I, trypsin-like and cathepsin L-like proteases), and Cys_2_His_2_ (C_2_H_2_) domain-containing zinc finger proteins [30,31,32]. Serpins may play a role in host immunoregulation [33], and endochitinases, and proteases such as cathepsin, may jointly participate in the microfilarial exsheathment process [34,35,36,37], whereas C_2_H_2_ domain-containing zinc finger proteins serve as transcription factors [38]. A quantitative and exploratory transcriptome profiling using dual RNA-sequencing (RNA-seq) chronicled the serial expression of *B. malayi* transcripts, spanning microfilarial stages to the development of human-infective larvae (L3) in the mosquito vector [39] (Table 1). Major findings from the study showed that transcriptome changes mediating cuticular moulting (transcripts encoding a number of regulatory and structural components) are pulsatile and that overall transcript expression oscillates between high levels during the intermittent period between two moults but is maintained at low levels during ecdysis [39].

Transcriptomic data on L3 from the mosquito showed upregulated expression of transcripts encoding various collagen protein family members on the cuticle, metabolic proteins, as well as proteins involved in stress resistance (e.g., dauer-enriched genes), pathogenesis, and immune resistance (e.g., serpin, cystatin, and abundant larval transcript–BmALT) and parasitisim (e.g., venom allergen-like protein–BmVAL-1) [31,32]. Those coding for the moult-mediating cathepsin L-like protease enzymes (e.g., BmCPL-1, BmCPL-4, and BmCPL-8) were also transcriptionally upregulated [31,32,36]. Generally, it is believed that the vast majority of these expressed proteins, in both mf and L3, are essential for establishment of the parasite infection and/or survival in the vector or in the subsequent mammalian host [31,32].

On the other hand, successful parasite transit through a vector indicates a degree of vector tolerability or susceptibility to the specific parasite, and the lack of this interspecies adaptive dialogue at the molecular or genetic level confers vector refractoriness to parasite invasion. The aim of manipulating susceptible phenotypes to drive refractoriness into the naturally susceptible vectors has motivated present research effort to develop particular interest in exploring the mosquito-expressed genes and gene products that confer susceptibility or refractoriness to filarial worms. Early investigators, who searched for the chromosomal regions of genes influencing *Ae. aegypti* susceptibility to filarial worms, found a sex-linked gene, ƒ*^m^*, located on chromosome 1 [40,41]. Subsequent molecular genetic linkage mapping approach using restriction fragment length polymorphism (RFLP) markers further identified two quantitative trait loci (QTL): fsb1 (corresponding to ƒ*^m^*) on chromosome 1 and an additive fsb2 on chromosome 2 [42]. Also, another QTL, idb2, which seems to influence *Ae. aegypti* ability to ingest *B. malayi,* was later found on chromosome 2 and is linked to the initial fsb2 [43].

Exploitation of the QTL to the causative gene(s) level has proved cumbersome, but recent exome sequencing, RNA-seq application, and an improved genetic linkage mapping using restricted-site-associated DNA (RDA) sequencing further highlighted that resistance of *Ae. aegypti* to *B. malayi* is driven by a single dominant sex-linked locus on chromosome 1 (corresponding to the QTL) and that this locus contains a number of known immune response genes, such as those controlling Toll, IMD, and JAK-STAT pathway activities, as well as other potential resistance-related genes [44,45]. Furthermore, studies have lent credence to early (usually within 48 hours) clearance of filarial worms by resistant mosquitoes with temporal expression of immune transcripts encoding antimicrobial peptides (cecropin and defensin) and transferrin, among others [45,46,47], while in susceptible mosquitoes, a filaria-induced upregulation of lipohorin and its receptor gene in the vector has been uncovered [48], an event that may facilitate parasite survival by suppressing mosquito immune responses [49,50,51].

The understanding of the physiological linkage between mosquito vectors and filarial parasites in relation to how the parasites cross their developmental checkpoints within the intermediate hosts has also been broadened. Transition of the ingested mf into L1 in mosquitoes occurs concurrently with an increased level of mosquito ecdysteroids, and this concomitant increase was also observed with initiation of L1 moulting to L2 and L2 to L3, implicating that ecdysteroid signaling is critical to the regulation of intramosquito filarial moulting [52]. Elucidating further the molecular trigger of moulting in filarial parasites, a functional *B. malayi* ecdysteroid receptor, Bma-EcR, was characterised [52].

## 3. Emerging Prospects of Achieving LF Eradication through Implementation of Mosquito–Parasite Approaches

Considering the vicious circle that may ensue with the current control of LF and the significant contribution from the insecticide-resistant disease-spreading vectors, knowledge of the molecular groundwork of the mosquito–filaria system and vector biology, coupled with the present technological advancement, has a great potential to translate into concrete ideas that may open wide avenues for developing new transmission-blocking or transmission-reducing strategies to combat LF more effectively. For instance, RNA interference (RNAi)-mediated silencing of the *Bmcpl-1* gene utterly disrupted *B. malayi* motility and development into the L3 stage in *Ae. aegypti* [53], indicating that robust understanding of the immunological, cellular, and physiological pathways or transductions in both filarial parasites and mosquitoes during the vector–parasite interface will direct research into devising novel transmission-blocking strategies, perhaps through delivery of transmission-blocking drugs (TBD) or vaccines (TBV) into the vectors. Drugs preventing mf exsheathment and mf migration through the mosquito gut wall, as well as those targeting the intramosquito developmental larval stages (L1, L2, and L3), have been proposed as potential targets for new antifilarial TBD designs [54]. For example, drugs acting as ecdysone or Bma-EcR antagonists may successfully arrest intramosquito filarial larval development. However, a potential but possibly circumventable difficulty in developing such drugs revolves around pharmacokinetics/pharmacodynamics (Pk/Pd) optimisation [55], regarding the inability to predetermine or influence the time frame between drug administration to patients and drug uptake by the haematophagous vectors in relation to the drug half-life, as well as the quantity of blood imbibed by the mosquitoes in relation to the volume of blood needed for effective drug action. Characterisation of the parasite- or vector-expressed surface molecules may also allow the isolation of potential transmission-blocking vaccine candidates [56]. The feasibility of this approach in curtailing transmission of mosquito-borne pathogens has been underpinned by the recent impressive progress made towards developing TBVs against *Plasmodium* parasites in their *Anopheles* vectors [57,58,59]. Altogether, TBD and TBV strategies will surpass the classical insecticide-based vector control, as they are not subject to selective pressure towards mosquito resistance [58]. Moreover, these strategies will be especially advantageous if conserved targets that exhibit broad-spectrum activity among different mosquito or parasite species or strains could be characterised, eliminating the need to develop targets for each mosquito–filaria combination.

Engineering of mosquitoes through transgenic technologies has become an increasingly emerging, mating-based control approach aimed at suppressing or modifying target vector populations in nature. Various methods employed in the current research activities are majorly based on sterile insect techniques (SITs) and gene drive systems [60,61,62]. SITs are self-limiting techniques that involve breeding and releasing of modified sterile males into the target area to mate with the wild females, and such mating results in the production of non-viable offspring [60]. Although the release of irradiated sterile male mosquitoes was recently tested in Sudan [63], Italy [64], and Indonesia [65], and showed encouraging performances, the recombinant DNA-based RIDL (release of insects carrying a dominant lethal gene) system [60,61,66], a spin-off of SIT, has been most successful and rapidly spreading in field trials. The most obvious evidence is the release of the transgenic OX513A strain of *Ae. aegypti* in the Caribbean [67,68], Malaysia [69] and Brazil [70], with encouraging outcomes towards suppressing target wild populations. In fact, after critical risk assessment, release of the OX513A strain was approved by the Brazilian National Technical Commission on Biosafety and will probably be executed nationwide in the near future [71]. An offshoot of the RIDL technique is also currently pursuing the production of flightless females in *Ae. aegypti* progeny [72,73], and experimental studies are likewise being undertaken using *Ae. albopictus* (Skuse) [74] and *An. stephensi* (Liston) [75].

The invasive, self-sustaining gene drive systems use selfish genetic elements to integrate and spread desired traits in a target mosquito species, either for the purpose of replacing existing wild mosquito populations with strains or species that are incapable of pathogen transmission by incorporating anti-pathogen effector genes into the vectors (modification strategy), or for the purpose of reducing or eliminating natural vector populations by driving detrimental genes into the populations (suppression strategy) [60,62,76]. The discovery of naturally-occurring selfish genetic elements, such as HEGs (homing endonuclease genes), the heritable *Wolbachia pipientis* (Hertig), and MEDEA (maternal effect dominant embryonic arrest), and the mechanisms of their activity, inspired the development of synthetic gene drive systems that have now become the major focus of the current research [60,62,76]. The new synthetic CRISPR/Cas9 (clustered regularly-interspaced short palindromic repeats/CRISPR associated protein 9) system overcomes many of the shortcomings of previous synthetic gene drives and is rapidly gaining ground in vector-borne disease research applications [62,76]. The natural genetic factors controlling mosquito resistance to filarial parasites can provide the basis for population modification strategies by delivering designed antifilarial RNA or peptide effector into mosquitoes through CRISPR/Cas9 using tissue-specific promoters [76], while introgression of desirable suppression genotypes into wild mosquito populations can also be executed by delivering Cas9/sgRNA (single guide RNA) complexes through embryo injection [62]. As with the antifilarial mosquito population modification, delivery of effector molecules into the thoracic flight muscles appears more attractive as the flight muscle-specific promoters have been identified [53,77], and the thoracic flight muscles provide the longest parasite exposure time to effector molecules, while the absorption of the molecules by the parasites may also be aided by cuticular lysis/turnover during moulting and protein uptake via the parasite gut [76]. Essential secreted proteins at the vector–parasite interface, such as those controlling neuromuscular activities and migratory behaviours, are potential targets for developing peptide effectors, whereas the functionally-characterised *Bmcpl-1* will serve in RNA effector applications [53,76].

The vast majority of current research activities towards the suppression of natural mosquito populations are being undertaken in the field of mosquito-borne viruses such as dengue, and in malaria control. However, a paramount benefit is the fact that mosquitoes acting as vectors for these diseases are also competent vectors of LF. Therefore, breakthroughs in these fields of research and eventual licensed application of the transgenic approaches on a global scale will have an immense synergistic effect in suppressing LF transmission, especially in areas where target mosquito species act as the major vector for LF or where the target mosquito-borne disease co-exists with LF. Moreover, particular species of transgenic mosquitoes can be released in LF-endemic areas even when other mosquito-borne diseases are not present.

## 4. Conclusions

The current speed in the war against mosquito-transmitted diseases is moving with an increasing energy. It is, however, clear that mosquito–filaria research has not been extensively explored, perhaps because some well-developed and advanced toolkits are still not in use. For instance, there are still indications that the genetic factors influencing mosquito resistance or susceptibility to filarial parasites have not been fully probed, but achieving this will require harnessing more advanced techniques and technologies [76]. Moreover, much remains to be done in the specific functional analysis of genes and proteins expressed in the intramosquito filarial stages. The quest to better understand these cardinal building blocks coordinating filarial parasites’ adaptation, survival, development, and other physiological activities within the vector will unveil hidden ‘golden nuggets’ required to design novel transmission-blocking chemotherapeutics, vaccines, and potent effector molecules for transgenic mosquito applications. Transgenic mosquito technologies have become a powerful tool for propelling vector control research in designing novel control methods with promising effectiveness; nevertheless, to gainfully utilize the potential of these emerging hi-tech control strategies, an effective dialogue with the public and the stakeholders, as well as resolving issues regarding the licensed deployment of engineered mosquitoes in nature, is crucial [78,79,80]. Overall, considerable progress has been made in the global control of LF, but achieving the projected elimination target is now clearly uncertain. To maintain the gains of the current drug-based, vaccine-lacking control approaches and the hoped effectiveness of the new MDA recommendations, co-implementation of the budding vector control and transmission-blocking concepts is essential for a synergistic control approach that will not only speed up LF elimination but also augment the potential for its future eradication.

## Figures and Tables

**Table 1:** Chronological RNA-seq expression profiles of *Brugia malayi* in *Aedes aegypti.*

Period	Upregulated Genes Ontology	Parasite Activity
Day 1–2	Mitochondrial ATP synthase complex, glycolysis, integral to membrane, DNA replication, signal peptidase complex, phosphoric diester hydrolase activity	Rearrangement and growth of preexisting microfilarial structure, extensive cuticular reorganisation, mf transforms into L1
Day 2–3	Calcium ion binding, response to stress, serine-type endopeptidase inhibitor activity, structural constituent of the cuticle	L1 development
Day 3–4	Ion channel activity, transmembrane transport, membrane, metallopeptidase activity, steroid hormone receptor activity	Middle to late L1 development: numerous mitotic divisions, lengthening of body, differentiation of internal structures, e.g., well-defined intestine
Day 4–5	Calcium ion binding, response to stress	First moulting into L2
Day 5–6	Serine-type endopeptidase inhibitor activity, structural constituent of the cuticle, metallopeptidase activity	L2 start to feed and develop: genital primordium is formed
Day 6–7	Glycolysis, integral to membrane, cysteine-type peptidase activity, structural constituent of the cuticle, steroid hormone receptor activity	L2 feed, elongate and further develop: rectum remains closed with anal plug
Day 7–8	Structural constituent of the cuticle, transmembrane transport, chloride transport	Second moulting into L3

Adapted from [39].
